# Physicochemical and Microbiological Properties of Synbiotic Yogurt Made with Probiotic Yeast *Saccharomyces boulardii* in Combination with Inulin

**DOI:** 10.3390/foods8100468

**Published:** 2019-10-10

**Authors:** Abid Sarwar, Tariq Aziz, Sam Al-Dalali, Xiao Zhao, Jian Zhang, Jalal ud Din, Chao Chen, Yongqiang Cao, Zhennai Yang

**Affiliations:** 1 Beijing Advanced Innovation Center for Food Nutrition and Human Health, Beijing Engineering and Technology Research Center of Food Additives, Beijing Technology and Business University, Beijing 100048, China; abiduomian@gmail.com (A.S.); iwockd@gmail.com (T.A.); salihsam4@gmail.com (S.A.-D.); zhaoxiao0828@163.com (X.Z.); zhangjian@btbu.edu.cn (J.Z.); jalal4827@gmail.com (J.u.D.); 2 Dongjun Dairy (Yucheng) Co., Ltd., Yucheng 251200, China; chaochen266@163.co (C.C.); caoyongqiang91@163.com (Y.C.)

**Keywords:** inulin, probiotics, prebiotics, synbiotic, *Saccharomyces boulardii*

## Abstract

*Saccharomyces boulardii* is a unique species of yeast previously characterized as a probiotic strain (CNCM I-745) among a few probiotic yeasts reported to date. Inulin is one of the most common prebiotics that exhibit twisted hydrocolloidal properties in dairy products. The present study was designed to develop a synbiotic yogurt by incorporation of *S. boulardii* and inulin at 1%, 1.5%, and 2% (w/v), comparing with the probiotic and control plain yogurts. Microrheological, microstructural, microbiological, sensory properties, and volatile compounds of the yogurt samples were evaluated. Microrheological analysis showed that addition of inulin to yogurt slightly reduced the values of G′ and G″, while solid–liquid balance (SLB) values confirmed more solid properties of the synbiotic yogurt (0.582~0.595) than the plain yogurt (0.503~0.518). A total of 18 volatile compounds were identified in the synbiotic yogurt, while only five and six compounds were identified in plain and probiotic yogurts, respectively. Physiochemical parameters such as pH, acidity, and protein content were in the normal range (as with the control), while fat content in the synbiotic yogurt decreased significantly. Addition of 1% inulin not only reduced syneresis but also maintained viability of *S. boulardii* after 28 days of storage. Microstructural and microrheological studies confirmed the dense, compressed, homogeneous structure of the synbiotic yogurt. Thus, addition of inulin improved the textural and sensory properties of the synbiotic yogurt, as well as survival of *S. boulardii* with viable count above 6.0 log CFU/g in yogurt, as generally required for probiotics. Therefore, novel synbiotic yogurt with desirable quality was developed as an effective carrier for delivery of the probiotic yeast exerting its beneficial health effects.

## 1. Introduction

Yogurt is a type of coagulated milk product with smooth texture, and that has a gentle sour and pleasant flavor that results from lactic fermentation with *Lactobacillus delbrueckii* ssp. *bulgaricus* and *Streptococcus thermophilus* [1]. Yogurt is the best-known nutritional carrier for efficient transfer of beneficial microbes into the body [2]. Recently there is an intensified demand for a new range of dairy products, including synbiotic yogurt containing both probiotics and prebiotics [3,4]. Probiotic lactic acid bacteria (LAB) have been shown to produce abundant bactericidal proteins in dairy foods [5,6]. Synbiotic yogurt has become increasingly popular as a type of functional food that beneficially affect the health condition of human beings [7,8].

Probiotics are live microbes that confer a health benefit for the host when introduced in a suitable amount [9]. Probiotic microorganisms are generally required to survive at body temperature and be resistant to stomach acid and bile salt [7]. Native bacteria are not probiotics unless they are isolated, purified, and proved beneficial to health when introduced. Prebiotics are a non-digestible part of food, which may serve as nutritional supplements for probiotic microorganisms to enhance their survival chances and implantation in the host intestinal tract [10]. Thus, prebiotics cause particular changes in both the composition and activity of the gastrointestinal microflora that confer benefits to the host well-being and health [11]. Probiotics may provide a potentially promising approach to preventing microbial dysbiosis [12]. However, synbiotics could better influence lipid profiles and protect against colorectal cancer than probiotics or prebiotics alone [13].

*Saccharomyces boulardii* CNCM I-745 was previously identified as a unique species of yeast characterized as a probiotic strain among a few probiotic yeasts reported to date [14]. Unlike other *Saccharomyces* strains with optimal growth at about 30 °C, *S. boulardii* survived best at 37 °C, which was advantageous as one of the few yeasts that did best at human body temperature [15]. *S. boulardii* was considered as a safe microorganism with non-toxic and non-pathogenic effects, and it could be implanted in large quantity in the gastrointestinal tract maintaining constant level of viability [16]. A bio-therapeutic agent based on the use of *S. boulardii* was developed by oral administration of this probiotic strain to treat recurrent *Clostridium difficile*-associated disease [17].

Inulin, one of the most common prebiotics, is mainly found in roots of chicory (*Cichorium intybus*), garlic (*Allium sativum*), wheat (*Triticum* spp.), oat (*Avena sativa*) and dalia [18]. It is known to be a storage polymer consisting of a β-2-1-linked fructosyl unit with a terminal glucosyl unit [19]. Currently, there is an increasing interest in addition of inulin and other oligofructose to food products (e.g., yogurt) for their healthful effects (e.g., enhancing *Lactobacillus* and *Bifidobacterium* growth in the colon, boosting bioavailability of a variety of minerals like calcium and iron, increasing antioxidant activities, and boosting immune functions) [20]. Inulin and oligofructose were shown to improve sensory quality and increase the probiotic viable count in functional dairy foods [21]. Supplementation of food fibers such as inulin could reduce wheying-off and thus improve the textural properties of food matrix, and it was also found to remarkably elevate viscosity and shear thinning behavior of different dairy products [22].

The present study was designed to develop synbiotic yogurt by using probiotic *S. boulardii* CNCM I-745 and prebiotic inulin for potential application in the dairy industry. Analysis and comparison of different yogurt samples containing this probiotic yeast and different concentration of inulin were performed in terms of changes of physicochemical, microbiological, and sensory properties, as well as microrheology and microstructure of the synbiotic yogurt throughout the cold storage period for 28 days. Attention was also paid to the effect of addition of the yeast and inulin on the syneresis of synbiotic yogurt resulted from exclusion of water as surface whey from the network of milk gel, leading to an adverse effect on perception of yogurt consumers.

## 2. Materials and Methods

### 2.1. Microorganisms and Culture Condition

Probiotic yeast (*S. boulardii* CNCM I-745) was purchased as a lyophilized powder in the form of sachet (Martin Dow, Karachi, Pakistan). The yeast culture (8.22 ± 0.28 CFU/mL) of *S. boulardii* was prepared according to the method described by Eunice et al. (2017), and used in yogurt making. The number of colony-forming units of *S. boulardii* (CFU/g) was determined on Sabouraud Dextrose agar in different dilutions made with dissolving a 250 mg sachet in 9 mL peptone water [23]. The yogurt starter culture containing *L. delbrueckii* ssp. *bulgaricus* and *Streptococcus thermophilus* was purchased in powder form (DANISCO, Sassenage, France), and activated by transferring consecutively for three times in 10% (w/v) reconstituted skim milk at 37 °C for 24 h. Inulin was purchased in powder form (Digestive-Now, USA).

### 2.2. Preparation of Yogurt

Fresh cow milk was purchased from a local dairy (SanYuan-Dairy Co., Ltd., Beijing, China). Five experimental groups of yogurt were arranged, including control plain yogurt (S1), probiotic yogurt with 0.5% *S. boulardii* (S2), synbiotic yogurt with 0.5% *S. boulardii* + 1% inulin (S3), 0.5% *S. boulardii* + 1.5% inulin (S4), 0.5% *S. boulardii* + 2% inulin (S5). The yogurt samples were made according to the method described in [23,24] with some modifications. After blending the fresh milk with 5% skim milk powder, the mixture was homogenized, pasteurized (85 °C, 30 min), and cooled to 43 °C. The mixture was inoculated with 3% (w/v) of the yogurt starter culture and mixed well. Then the *S. boulardii* culture and inulin were added. The yogurt samples were incubated at 43 ± 2 °C until about pH 4.5, and then they were stored at 4 °C for four weeks. Sampling was performed every week during the storage for the following analyses.

### 2.3. Physiochemical Parameters

Physiochemical parameters of yogurt samples during the cold storage were determined using standardized instrumental methods [25]. The pH was determined with a Metrohm pH meter (Metrohm AG, Herisau, Switzerland) at room temperature. Total solids, total fat, and titratable acidity of the samples at different storage times were determined as described [26]. Syneresis of the yogurt samples at different storage times was determined as described [27]. Briefly, yogurt samples (30 g) were centrifuged at 1677× *g* for 10 min, and then the supernatant was poured off, weighed, and recorded as a percentage syneresis.

### 2.4. Texture Profile Analysis of Yogurt Samples

Texture profile analysis of the yoghurt samples was done by using a texture analyzer (Brook-Field Texture Analyzer, CT3 1000, Middleboro, MA, USA) with a 5 kg load cell on day 0. Each yogurt sample was placed in a 100 mL plastic cup for compression test. The probe used was cylindrical with a rounded edge and 20 mm in length. Two cycles were applied, at a constant crosshead velocity of 1 mm s^−1^, to a sample depth of 20 mm, with a surface trigger of 5 g. Hardness, cohesiveness, adhesiveness, stickiness, and gumminess of the yogurt samples were tested in three replicate batches.

### 2.5. Microbial Enumeration

The microbiological analysis of the yogurt samples was performed during the cold storage. The pour plate technique was used to determine total bacterial count (TBC) and total yeast count (TYC). Briefly 1 g of yogurt sample was diluted in 9 mL of peptone water. For lactic acid bacterial viable counting, MRS (De Man, Rogosa and Sharpe-Oxoid, Basingstoke, Hants, UK) agar medium was used; and for yeast, Sabouraud dextrose agar (HiMedia, PA, USA) was used. The enumeration of bacterial count was done after 24 h of incubation at 37 °C and for yeast count it was done after incubation for 72 h at 37 °C.

### 2.6. Volatile Analysis

The headspace solid-phase microextraction coupled with gas chromatography-mass spectrometry (HS-SPME-GC-MS) was used to analyze the volatile compounds in the yogurt samples on day 0 of storage. A 20 mL head-space vial was used to mix 5 g of sample with 10 µL of 1,2-dichlorobenzene as internal standard (I.S, 800 ppm), then tightly covered by a silicon septum. The extraction was done by the fiber made from StableFlex divinylbenzenecarboxen polydimethylsiloxane (DVB-CAR-PDMS) in 50/30 µm (Supelco, Bellefonte, PA, USA), in a water bath at 70 °C for 60 min. Then, the absorbed volatiles were desorbed in injection port of GC-MS at 250 °C for 5 min in splitless mode.

Analysis by GC (Agilent 7890B, Santa Clara, CA, USA) coupled with MS (Agilent 7200, Santa Clara, USA) was performed using a semi- non-polar column (HP-5MS, 30 m × 0.25 mm × 0.25 µm, Agilent Technology, USA) with helium as the carrier gas at a flow rate of 1 mL/min. The initial oven temperature was kept at 60 °C for 2 min, then ramped at 7 °C/min to 250 °C and maintained to 30 min. The MS detector was operated in an electron ionization (EI) voltage of 70 eV under a mass scan range of 33 to 450 amu (m/z). The temperature of both ion source and line transfer was set at 250 °C.

Quantitation analysis was done by using the internal standard method, where the peak area for the identified volatiles was compared to their corresponding peak area of the internal standard (i.e., 1,2-dichlorobenzene). The concentration of each compound was calculated by the following equation:

Concentration of volatiles = (peak area of the compound/peak area of the internal standard) × concentration of the internal standard.(1)

All the samples were analyzed in triplicate.

### 2.7. Sensory Evaluation

Sensory analysis of the yogurt was performed by a panel of 12 judges, who were trained and familiar with yogurt attributes, on days 0, 7, 14, 21, and 28 of the storage period. Yogurt samples were coded with numbers and presented together to panel members in daylight. Water was provided for rinsing of the mouth after each sample. Sensory parameters such as color and appearance, taste and odor, texture and overall acceptability were rated on a 9-point hedonic scale (scoring 1—dislike extremely to 9—like extremely. The obtained sensory scores were statistically evaluated by means of analysis of variance (ANOVA) using two factor randomized design. The significant differences among the means were determined by applying the least significance difference (LSD).

### 2.8. Microstructural Analysis

The microstructure of the yogurt samples was studied using a method described by Yen et al. [28] with slight modification. The yogurt samples were prepared for scanning electron microscopy by taking 0.3 g yogurt (1 cm below the surface) and mixing with 0.3 g of 3% aqueous agar solution at 45 °C. After solidification, the gelled samples were cut into 1 mm cubes and fixed in 2% glutaraldehyde solution in phosphate buffer at room temperature for 2 h, and then at 4 °C for 24 h. The samples were washed again with phosphate buffer and dehydrated for 15 min in a graded ethanol series consisting of 50%, 70%, 90%, and 100% ethanol. The samples were then frozen in liquid nitrogen. The dried samples were mounted on aluminum SEM stubs by using a carbon-based tape and coated with gold in ES-1010 sputter coater (Hitachi, Tokyo, Japan).

### 2.9. Microrheological Analysis

The microrheology analysis of the yogurt samples (20 mL) was performed by using Rheolaser Master (Passive µRheology, Formulaction, Toulouse, France). The temperature was set at 25 °C for all the samples. Mean square displacement (MSD) slopes, storage modulus (G′), and loss modulus (G″) were measured and recorded.

### 2.10. Statistical Analysis

All measurements were done in triplicate. The results were statistically analyzed by one way ANOVA by using Statistix 8.1 software (Analytical software, 2105 MillerLanding Rd, Tallahassee, FL, USA).

## 3. Results and Discussion

### 3.1. Physiochemical Parameters of Synbiotic Yogurt

The physiochemical parameters of the yogurt samples before cold storage are shown in Table 1. All samples were moved to cold storage when the pH dropped to 4.5 (commonly practiced pH level in yogurt production), and there was no significant difference (*p* > 0.05) among all the samples in terms of the incubation time needed to reach this pH level. Titratable acidity of the yogurt samples before cold storage were from 0.87% to 0.90%. Fat content of the control sample S1 (3.11%) was almost the same as that (3.10%) of the probiotic yogurt S2, but the fat content of the synbiotic yogurt S5 (3.05%) was slightly lower due to addition of inulin. Addition of inulin also slightly increased total solid contents in the synbiotic yogurt samples (S3, S4, and S5), while the protein contents of all the samples before storage had no significant differences (*p* > 0.05).

**Figure 1:**
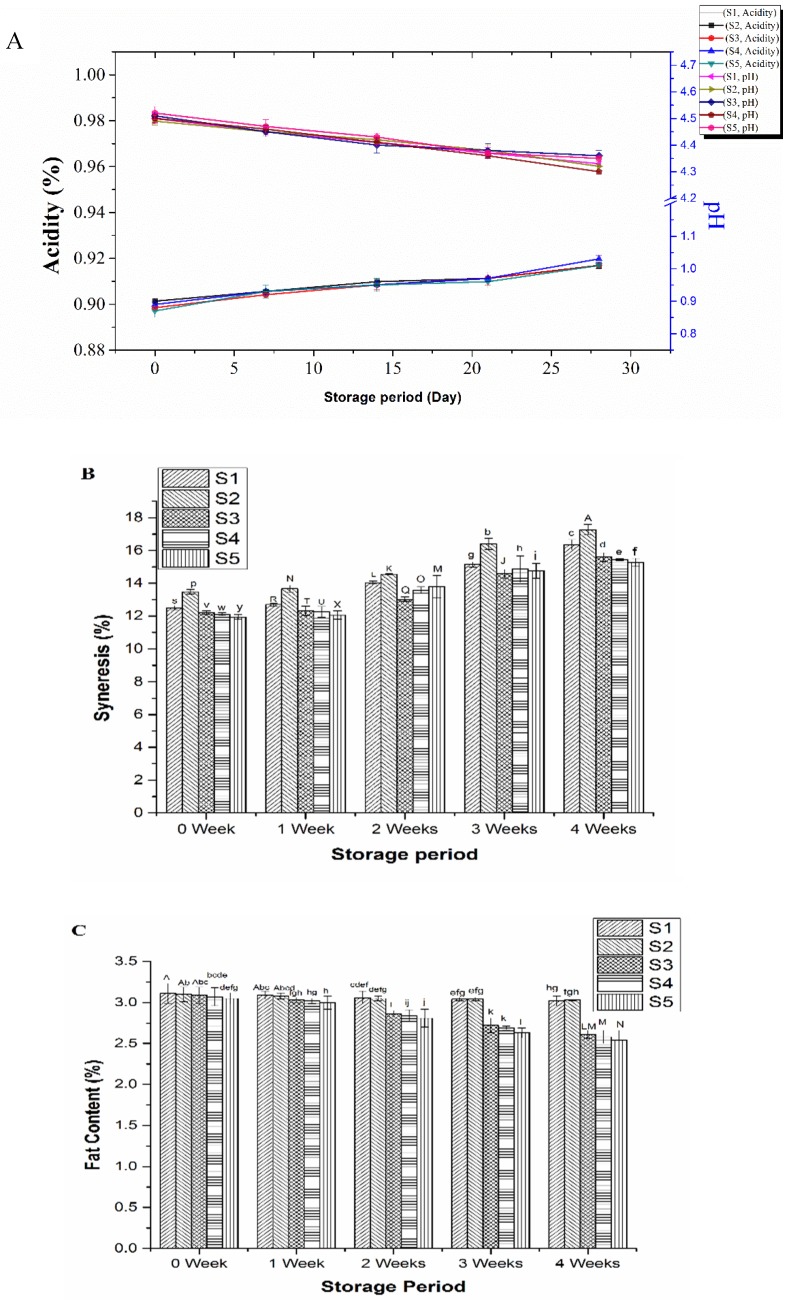
Change of pH and acidity (**A**), syneresis (**B**), and fat content (**C**) of the yogurt samples during storage at 4 °C for 28 days: Control plain yogurt (S1), probiotic yogurt with 0.5% *S. boulardii* (S2), synbiotic yogurt with 0.5% *S. boulardii* + 1% inulin (S3), 0.5% *S. boulardii* + 1.5% inulin (S4), 0.5% *S. boulardii* + 2% inulin (S5). Different letters denote statistical differences (*p* < 0.05).

Changes of physiochemical parameters of yogurt including pH, acidity, fat, and protein content during four weeks of storage at 4 °C are shown in Figure 1. The pH of all the yogurt samples decreased slightly in the storage period, while the titratable acidity increased, ranging from 0.92 to 1.03, during the storage (Figure 1A). Addition of inulin at different concentrations and *S. boulardii* had no significant (*p* > 0.05) effect on the change of pH or acidity of yogurt, suggesting that both the prebiotic and yeast did not alter the capacity of acid production by the starter LAB in yogurt. Previously, no significant effect of the addition of inulin on the pH of yogurt was also observed, though there was slightly more decrease in pH of yogurt during the third and fourth weeks of storage [29]. The increased acidity of yogurt during cold storage could be due to microbial activity and enzymes produced during fermentation that promoted conversion of residual carbohydrates (mainly lactose) to lactic acid, CO_2_, and formic acid [11,30,31,32,33,34]. Syneresis was considered a leading problem during the prolonged storage of yogurt, and separation of the released whey from the shrank gel part of yogurt deteriorated the texture and mouthfeel of the product [11,28]. Addition of inulin obviously decreased syneresis of the yogurt with less syneresis at higher concentration of inulin because of the water-holding capacity of inulin (Figure 1B).

Regarding the compositional changes of the yogurt during cold storage, though addition of inulin and *S. boulardii* did not affect significantly the protein and total solid contents of yogurt, the fat content decreased significantly (*p* < 0.05) during the later stage of cold storage (Figure 1C). A similar decrease in fat content of yogurt was also reported earlier by Guven et al. [29] when inulin was used for making yogurt. However, whether inulin played a role in the metabolism of fat by *S. boulardii* leading to decreased fat content in yogurt needs to be further studied.

### 3.2. Texture Profile

**Figure 2:**
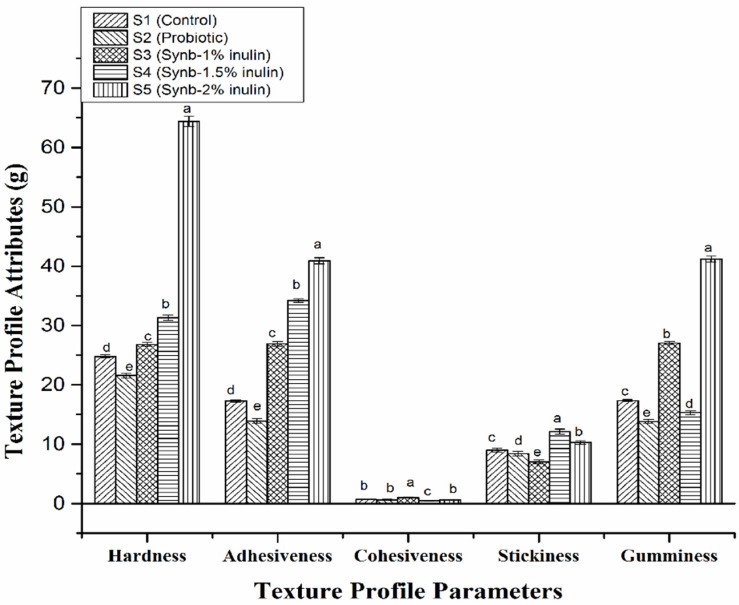
Texture profile analysis of the control plain yogurt (S1), probiotic yogurt with 0.5% *S. boulardii* (S2), synbiotic yogurt with 0.5% *S. boulardii* + 1% inulin (S3), 0.5% *S. boulardii* + 1.5% inulin (S4), and 0.5% *S. boulardii* + 2% inulin (S5). Different letters denote statistical differences (*p* < 0.05).

Texture profile analysis of different yogurt samples was carried out on the last day of storage and the results are presented in Figure 2. The textural parameters (i.e., hardness, adhesiveness, cohesiveness, stickiness, and gumminess) showed significant changes with the addition of different concentration of inulin and *S. boulardii* compared with the control yogurt. Addition of *S. boulardii* with starter culture decreased hardness in the probiotic yogurt S2, probably because of fermentation of available sugars by the yeast. However, addition of different concentrations of inulin increased the hardness (S3 from 24.80 to 26.80 g, an 8% increase; S4 from 24.80 to 31.30 g, a 26% increase; and S5 from 24.80 to 64.40, a 159% increase). Use of inulin increased the viscosity of yogurt samples by binding and orienting water that did not integrate into the protein network and inhibited wheying-off resulting in strong casein micelles aggregation. The same pattern was observed in terms of adhesiveness in probiotic and synbiotic yogurt samples. Cohesiveness of different yogurt samples was in the range from 0.49 to 1.01. Surprisingly, the highest cohesiveness was observed in S3 where 1% inulin was used. Stickiness and gumminess also showed significant variation among the samples compared with the control. The use of inulin increased gumminess but there was no particular trend.

Mudgil et al. [35] added gelatin to camel milk yogurt and found a three-fold increase with 1% gelatin, and a seven-fold increase with 1.5% gelatin. Pang et al. [36] used 1% and 2% starch that significantly increased firmness and adhesiveness of yogurt. Mariano et al. [37] studied different thickening agents such as starch and whey protein, and reported increased values in the texture profile of yogurt. Analogous results were reported by Sandoval-Castilla et al. [38] with modified tapioca starch. Supavititpatana et al. [39] also reported improvement in hardness, springiness, and adhesiveness of corn yogurts produced by addition of gelatin. From the texture profile analysis described above, our study indicated that the synbiotic yogurt with 1% inulin exhibited better textural properties, in terms of hardness, adhesiveness, and gumminess, without negatively affecting the palatability of natural yogurt in comparison with the control and probiotic yogurts. Thus, addition of probiotic (*S. boulardii)* alone and with combination of prebiotic (inulin) in different concentrations potentially influenced the texture properties of yogurt.

### 3.3. Survivability of S. boulardii and LAB in Synbiotic Yogurt

**Figure 3:**
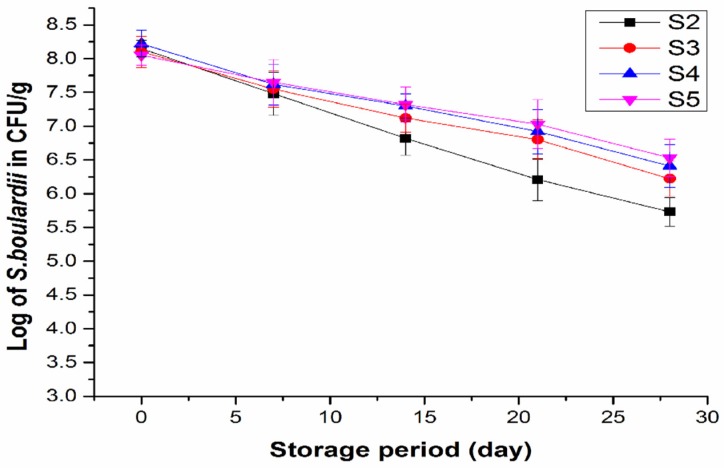
Survival of *S. boulardii* in the yogurt samples during storage at 4 °C for 28 days: Probiotic yogurt with 0.5% *S. boulardii* (S2), synbiotic yogurt with 0.5% *S. boulardii* + 1% inulin (S3), 0.5% *S. boulardii* + 1.5% inulin (S4), 0.5% *S. boulardii* + 2% inulin (S5).

The survivability of *S. boulardii* as affected by inulin at different concentrations during storage of yogurt at 4 °C is presented in Figure 3. The viable counts (≥8 log CFU/g at day 0 of storage) of the yeast in the yogurt samples S2, S3, S4, and S5 significantly decreased (*p* ≤ 0.05) throughout the four weeks’ storage period. Higher viable counts of the yeast in the yogurt samples the added inulin, than that in the sample without inulin, were observed after 14 days of storage, suggesting that inulin played a role in maintaining the viability of the yeast in yogurt. In the sample (S2) without inulin, the viable count of the yeast decreased from initial ≥8 to 5.5 log CFU/g at day 28, while it was higher than 6 log CFU/g for S3 (6.22 log CFU/g), S4 (6.41 log CFU/g), and S5 (6.53 log CFU/g) with inulin at the end of the storage, satisfying the minimum requirement of the Food and Agriculture Organization (FAO) and World Health Organization (WHO) guidelines for probiotics [9]. Viability of probiotics was reported to be affected by many factors such as storage time, oxygen content, fluctuation in temperature, low pH, reduced water activity, and high concentration of salutes [40,41]. Microencapsulation method was applied for protection of *S. boulardii* to increase its survival, but this technique increased the production cost [23]. The present study employing inulin provided an effective and economic approach in the development of synbiotic yogurt containing the probiotic yeast with enhanced viability.

The viable counts of LAB in all yogurt samples showed slight decreases during the 28 days of storage. At day 0 the viable counts of LAB for S1, S2, S3, S4, and S5 samples were 8.62, 8.41, 8.35, 8.14, and 8.06 log CFU/g, respectively. Whereas, at day 28 they decreased to 7.43, 7.37, 7.29, 7.17, and 7.10 log CFU/g for S1, S2, S3, S4, and S5, respectively. Eunice et al. [23] also showed good viability of LAB when *S. boulardii* was added to yogurt.

### 3.4. Volatile Compounds of Synbiotic Yogurt

Flavor is often the first indicator when consumers choose food. The consumers do not take interest in functional food consumption if biologically active ingredients lead to an unpleasant flavor [30]. Volatile analysis is widely applied in the objective assessment of dairy foods to determine the acceptance of new functional products by consumers [42]. Formation of volatile compounds in the synbiotic yogurt made with probiotic *S. boulardii* and inulin, as compared to the control plain yogurt and the probiotic yogurt with the yeast without inulin, is shown in Table 2. Analysis by HS-SPME-GC-MS showed that a total of 18 volatile compounds were identified in the synbiotic yogurt, while only five and six compounds were identified in the plain and probiotic yogurts, respectively. These volatile compounds were from different chemical families including two aldehydes (3-furaldehyde and 5-hydroxymethylfurfural), three acids (acetic acid, hexanoic acid, and octanoic acid), six esters (formic acid, isopentyl ester, butanoic acid-3-methyl-3-methylbutyl ester, octanoic acidmethyl ester, octanoic acidethyl ester, decanoic acidethyl ester, and hexadecanoic acidethyl ester), two ketones (2,4-dihydroxy-2,5-dimethyl-3(2H)-furan-3-one and 2,3-dihydro-3,5-dihydroxy-6-methyl-4H-pyran-4-one), four alcohols (3-furanmethanol, phenylethyl alcohol, maltol, and 2-(2-butoxyethoxy)-ethanol), one pyrazole (3-methoxycarbonylpyrazole), and other two miscellaneous compounds (methoxyphenyl oxime and naphthalene). The concentration of these compounds ranged from 18.01 µg/L (2-(2-butoxyethoxy)-ethanol)) to 305.09 µg/L (hexanoic acid). The total concentration of the volatile compounds formed in the yogurt samples was in the order of S4 (1434.15 µg/L), S3 (1361.58 µg/L), S5 (948.58 µg/L), S2 (229.69 µg/L), and S1 (134.77 µg/L). Therefore, addition of inulin and the yeast significantly increased the amounts and types of volatile compounds in yogurt. This suggests the possible mechanism of prebiotic inulin to modify the metabolism of the probiotic yeast and LAB in the formation of volatiles, which needs to be further studied.

Aldehydes are considered as important aroma compounds contributing to the volatile profile of fermented dairy products with lactic acid bacteria [43,44]. In the synbiotic yogurt of this study, 5-hydroxymethylfurfural (115.41 µg/L) and 3-furaldehyde (about 50 µg/L) were detected at relatively high levels. Acid compounds were generally present in various fermented dairy products [45,46]. Among the three acid volatiles identified, hexanoic acid was found in all the yogurt samples at a concentration ranging from 26.27 µg/L (S1) to 305.09 µg/L (S4), while octanoic acid was detected in the yogurt samples (S1, S2) without inulin, and acetic acid only in one synbiotic yogurt sample (S3). Ester volatiles were generally produced at low concentration in dairy products when lactose was fermented by LAB [47]. Octanoic acid ethyl ester and hexadecanoic acid ethyl ester were detected in all the yogurt samples of this study, but formic acid isopentyl ester was detected only in the probiotic yeast yogurt. Other three esters were found in the symbiotic yogurt samples. Ketone compounds play a key role in the creamy flavor of dairy products [48]. Two ketone compounds were found only in synbiotic yogurt samples of this study. Alcoholic compounds also impart their contribution in flavor improvement in fermented dairy products. A total of four alcohols were identified in all the synbiotic yogurt samples. Formation of more volatile compounds in the synbiotic yogurt samples enriched their flavor, compared to the plain and probiotic yeast yogurts that contained less volatiles. Dan et al. [49] also showed that the aroma profiles of yogurt made with pure culture were different from those made with addition of probiotics alone or with combination of prebiotics.

### 3.5. Sensory Evaluation

The sensory property of the yogurt samples was evaluated in terms of color and appearance, taste and odor, texture and overall acceptability, as shown in Table 3. Compared with the control (S1) plain yogurt, the synbiotic yogurt with 1% inulin (S3) had higher sensory scores for color and appearance, taste and odor. As reported by Golob et al. [50], addition of inulin in dairy products improved mouthfeel and taste. Although addition of the probiotic yeast *S. boulardii* decreased the scores for the texture of yogurt, production of alcohol and carbon dioxide (CO_2_) contributed to the enhanced flavor and taste. Wang et al. [51] reported that carbon dioxide (CO_2_) and alcohol produced by yeast contributed to the refreshing and foamy taste of Kefir. Furthermore, addition of inulin in S3, S4, and S5 recovered the textural scores of yogurt gradually. However, inulin at higher concentration (2%) decreased the sensory scores for the taste, odor, and overall acceptability of synbiotic yogurt S5. Inulin was reported to provide creamy mouthfeel and sweet taste to yogurt [52]. Carbohydrate fat substitutes such as inulin in yoghurt production improved perception of color [53], and this would help overcome the problem of slight discoloration of synbiotic yogurt during the fourth week of storage, as observed in this study. Bano et al. [54] also reported that color of functional yogurt could be significantly affected by storage time. Overall the synbiotic yogurt supplemented with 1% inulin (S3) possessed desired sensory properties. There were no significant differences among S1, S3, and S4 in overall acceptability, but S2 and S5 had relatively low acceptability.

### 3.6. Microstructure and Microrheology of Synbiotic Yogurt

**Figure 4:**
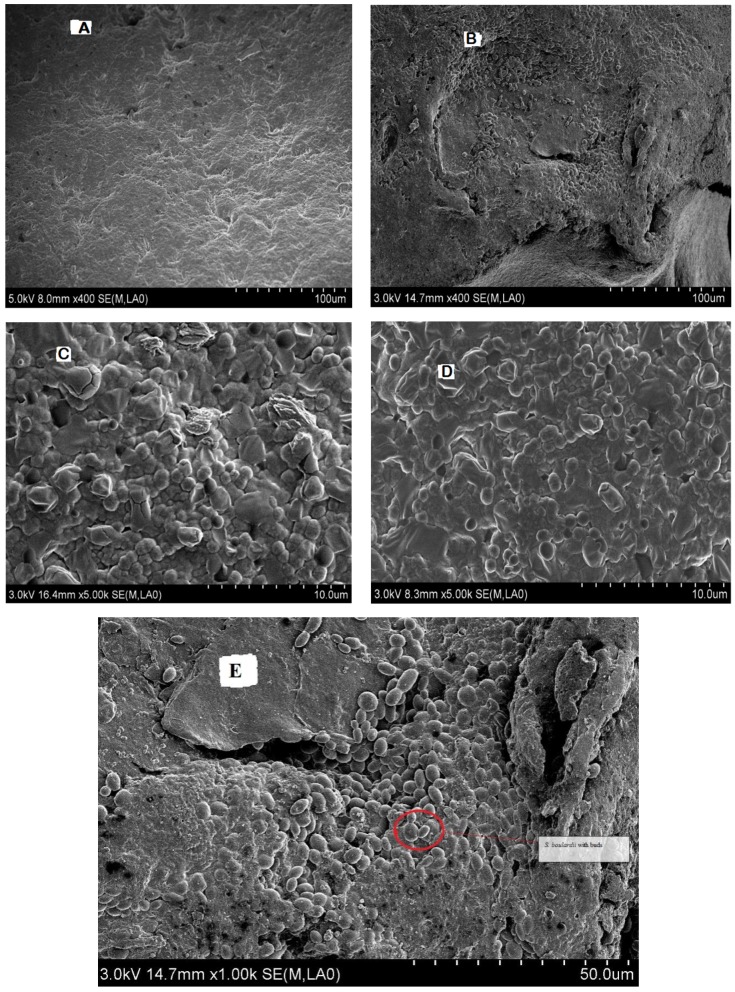
Scanning electron microscope images at low (400×) and high (5000×) magnifications of the control plain yogurt sample S1 (**A**,**C**) and synbiotic yogurt sample S3 with 0.5% *S. boulardii* + 1% inulin (**B**,**D**,**E**).

The microstructure of the desirable synbiotic yogurt (S3) was further studied in comparison with the control plain yogurt (S1) by scanning electron microscope. As shown in Figure 4B,D, the synbiotic yogurt exhibited a more dense, compressed, and homogeneous microstructure with no or negligible spaces, and the whey was firmly restrained in the gel matrix. The *S. boulardii* cells were also visible with buds, confirming their viable and multipliable state at the end of storage (Figure 4E). In contrast, the plain yogurt showed an irregular branched network with wide spaces (Figure 4A,C). Previously, incorporation with gelatin in camel milk yogurt also resulted in a more compact and homogeneous protein network structure with reduced syneresis, mainly due to inter- and intra-molecular polymeric interactions in the yogurt [35,39].

**Figure 5:**
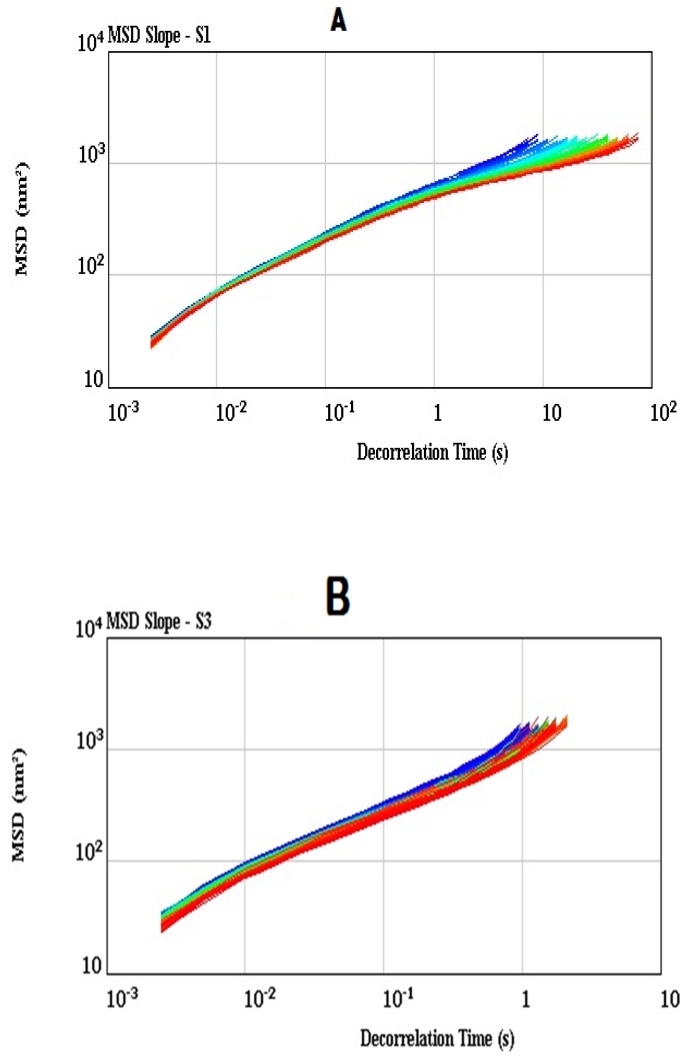
Mean square displacement (MSD) (**A** for S1, **B** for S3).

**Figure 6:**
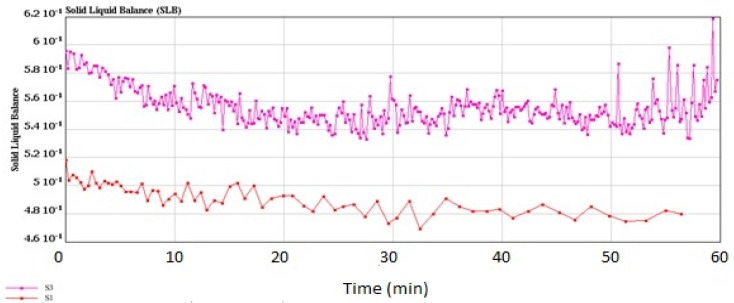
Solid–liquid balance (SLB) values of the control plain yogurt (S1) and synbiotic yogurt with 0.5% *Saccharomyces boulardii* + 1% inulin (S3).

**Figure 7:**
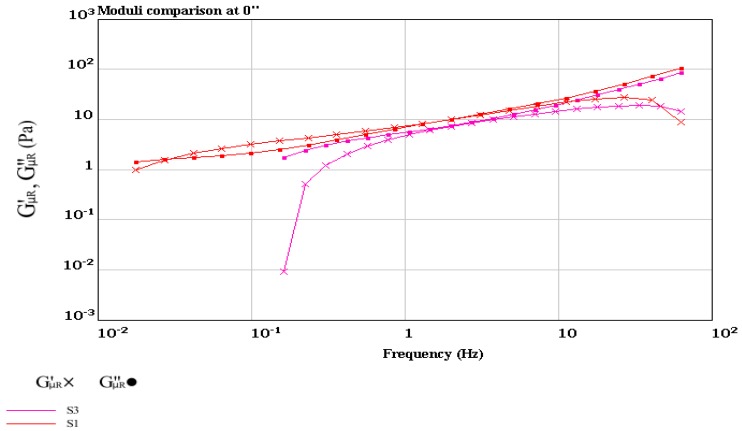
Storage modulus (G′) and loss modulus (G″) of the control plain yogurt (S1) and synbiotic yogurt with 0.5% *Saccharomyces boulardii* + 1 % inulin (S3).

Microrheology measures local deformation of a sample resulted from an applied stress or due to thermal energy by using micron-sized particles dispersed in a liquid [55]. The microrheological properties of the synbiotic yogurt (S3) as compared to the control plain yogurt (S1) were studied, as shown in Figure 5, Figure 6 and Figure 7. The MSD values of both the synbiotic yogurt (Figure 5A) and plain yogurt (Figure 5B) increased almost linearly with the decorrelation time, suggesting the viscous properties of both the yogurt samples. Similar linear increase in MSD values indicating the purely viscous characteristics of the whey protein concentrate when added from 40% to 80% oil was also reported [56]. However, the synbiotic yogurt due to addition of inulin exhibited a more viscous nature than the plain yogurt, as indicated by the shorter decorrelation time (1 s) of the former than that (10 s) of the latter reaching the MSD value of about 1000 nm^2^. Changes of MSD values provided Brownian motion information in the yogurt samples [57]. The MSD slop value of less than 1 indicated that the motion of the particles or droplets is blocked; the value of 1 illustrating a Brownian motion type, and value of greater than 1 illustrating a ballistic motion type [58].

Determination of the solid–liquid balance (SLB) values of the yogurt samples confirmed more solid properties of the synbiotic yogurt than the plain yogurt, since the former had higher SLB values (0.582~0.595) than the latter (0.503~0.518) (Figure 6). The SLB values above 0.50 indicated transformation from liquid domination to solid domination, while the SLB value of 0.5 was the critical value of a balanced state of liquid and solid [59]. Determination of the storage modulus (G′) and loss modulus (G″) of the yogurt samples at the frequency range of 10^−3^ to 10^2^ Hz (Figure 7) showed that addition of inulin to yogurt slightly reduced the values of G′ and G″, as reported by Pang et al. [36] when starch was added in acid milk gels. Polysaccharides such as inulin had associative and segregative interactions with milk proteins [60,61]. Thus, addition of inulin in yogurt making could induce earlier gelation resulting from uptake of water by the inulin granules during swelling.

## 4. Conclusions

Probiotic yeast *S. boulardii* and prebiotic inulin were fortified in synbiotic yogurt and inulin played a role in the improvement of the texture, taste, and mouthfeel, and the decreased syneresis of yogurt during cold storage. Addition of inulin did not affect the growth of the yogurt starter LAB, and maintained survival of *S. boulardii* with viable count of more than 6.0 log CFU/g in yogurt, satisfying the minimum requirement of the FAO and WHO guidelines for probiotics. Combination of both inulin and the yeast positively influenced textural attributes such as hardness, cohesiveness, and adhesiveness, but use of the yeast alone reduced hardness. This functional yogurt (S3) containing more favorable volatile compounds (1434.15 µg/L) than the control plain yogurt (134.77 µg/L) could heighten consumer acceptability, representing a novel synbiotic dairy product with probiotic yeast. Microstructure and microrheology results confirmed the dense, compressed, homogeneous structure of S3 compared with the control yogurt.

Therefore, a synbiotic yogurt employing inulin and *S. boulardii* with desirable quality was developed in this study, for potential application as a novel functional dairy product with beneficial health properties.

## Figures and Tables

**Table 1:** Physiochemical parameters of yogurt samples before cold storage.

Samples	pH	Acidity (%)	Fat (%)	Total Solid (%)	Total Protein (%)
S1	4.50 ± 0.04 **^a^**	0.89 ± 0.05 **^a^**	3.11 ± 0.12 **^a^**	16.48 ± 0.13 **^b^**	3.71 ± 0.07 **^a^**
S2	4.49 ± 0.07 **^a^**	0.90 ± 0.03 **^a^**	3.10 ± 0.09 **^a^**	16.45 ± 0.15 **^b^**	3.49 ± 0.04 **^d^**
S3	4.51 ± 0.05 **^a^**	0.88 ± 0.07 **^a^**	3.09 ± 0.10 **^a^**	16.57 ± 0.12 **^ab^**	3.55 ± 0.06 **^cd^**
S4	4.50 ± 0.02 **^a^**	0.89 ± 0.04 **^a^**	3.07 ± 0.11 **^a^**	16.64 ± 0.10 **^ab^**	3.59 ± 0.09 **^bc^**
S5	4.52 ± 0.08 **^a^**	0.87 ± 0.08 **^a^**	3.05 ± 0.07 **^a^**	16.83 ± 0.18 **^a^**	3.65 ± 0.05 **^ab^**

S1—control plain yogurt; S2—probiotic yogurt with 0.5% *S. boulardii*; S3—synbiotic yogurt with 0.5% *S. boulardii* + 1% inulin; S4—0.5% *S. boulardii* + 1.5% inulin; S5—0.5% *S. boulardii* + 2% inulin. Different letters superscripted denote statistical difference (*p* < 0.05) within a column.

**Table 2:** Volatile compounds of the yogurt samples identified by HS-SPME-GC-MS.

						Concentration (µg/L)			
Volatiles	RT ^a^	RI ^b^	CAS	Identification	S1	S2	S3	S4	S5
**Acids**									
Acetic acid	2.05	610	64-19-7	RI, MS	n.d	n.d	88.19	n.d	n.d
Octanoic acid	12.18	1178	124-07-2	RI, MS	28.41	25.80	n.d	n.d	n.d
Hexanoic acid	6.35	937	142-62-1	RI, MS	26.27	51.13	80.66	305.09	46.26
**Esters**									
Formic acid, isopentyl ester	2.39	831	110-45-2	RI, MS	n.d	99.30	n.d	n.d	n.d
Butanoic acid, 3-methyl-, 3-methylbutyl ester	9.82	1105	659-70-1	RI, MS	n.d	n.d	n.d	n.d	29.09
Octanoic acid, methyl ester	10.42	1123	111-11-5	RI, MS	n.d	n.d	n.d	22.93	20.22
Octanoic acid, ethyl ester	12.8	1197	106-32-1	RI, MS	34.48	53.46	101.09	207.18	180.43
Decanoic acid, ethyl ester	19.2	1395	110-38-3	RI, MS	n.d	n.d	n.d	n.d	29.01
Hexadecanoic acid, ethyl ester	35.43	2093	628-97-7	RI, MS	loq	loq	loq	42.37	16.40
**Aldehydes**									
3-Furaldehyde	3.41	835	498-60-2	RI, MS	n.d	n.d	50.57	51.43	48.52
5-Hydroxymethylfurfural	13.85	1224	67-47-0	RI, MS	n.d	n.d	115.41	n.d	n.d
**Alcohols**									
3-Furanmethanol	3.85	864	4412-91-3	RI, MS	n.d	n.d	80.89	85.54	82.12
Phenylethyl alcohol	10	1110	60-12-8	RI, MS	n.d	n.d	128.23	75.12	51.70
Maltol	10.13	1115	118-71-8	RI, MS	n.d	n.d	62.50	70.33	77.12
2-(2-Butoxyethoxy)- ethanol	12.52	1188	112-34-5	RI, MS	n.d	n.d	n.d	20.32	18.01
**Ketones**									
2,4-Dihydroxy-2,5-dimethyl-3(2H)-furan-3-one	6.3	989	10,230-62-3	RI, MS	n.d	n.d	84.78	80.09	78.22
2,3-Dihydro-3,5-dihydroxy--6-methyl-4H-pyran-4-one	11.16	1146	28,564-83-2	RI, MS	n.d	n.d	258.34	21.92	n.d
**Pyrazole**									
3-Methoxycarbonylpyrazole	9.16	1083	15,366-34-4	RI, MS	n.d	n.d	82.43	85.44	90.23
**Others**									
Methoxy-phenyl- oxime	4.32	…	…	MS	45.61	loq	228.48	229.47	40.80
Naphthalene	12.2	1178	91-20-3	RI, MS	n.d	n.d	n.d	136.91	140.44

^a^ RT—retention time; ^b^ RI—retention index was determined with HP-5 column by injection of a mix on *n*-alkane (c6–c23). MS, mass spectra; S1—control plain yogurt, S2—yogurt with *S.* *boulardii*, S3—yogurt with *S.* *boulardii* + 1% inulin, S4—yogurt with *S.* *boulardii* + 1.5% inulin, S5—yogurt with *S.* *boulardii* + 2% inulin, n.d—not detected, loq—low of quantitation.

**Table 3:** Mean sensory scores with standard deviation given by panelists (n = 12) for each yogurt sample on a hedonic scale of nine-points.

Sample	Color and Appearance					Taste and Odor					Texture					Overall Acceptability				
	Storage/Day					Storage/Day					Storage/Day					Storage/Day				
	0	7	14	21	28	0	7	14	21	28	0	7	14	21	28	0	7	14	21	28
**S1**	$7.90{\;}^{h}\pm 1.05$	$7.78{\;}^{l}\pm 1.1$	$7.69{\;}^{n}\pm 1.12$	$7.45{\;}^{r}\pm 1.50$	$7.30{\;}^{t}\pm 1.28$	$8.30{\;}^{b}\pm 1.15$	$8.21{\;}^{e}\pm 1.21$	$8.10{\;}^{g}\pm 1.1$	$7.87{\;}^{j}\pm 1.5$	$7.73{\;}^{m}\pm 0.9$3	$7.30{\;}^{a}\pm 1.11$	$7.20{\;}^{c}\pm 1.21$	$7.09{\;}^{e}\pm 1.41$	$7.0{\;}^{f}\pm 1.1$	$6.84{\;}^{i}\pm 1.31$	$8.42{\;}^{a}\pm 1.24$	$8.36{\;}^{\mathrm{ab}}\pm 1.18$	8.14 ^cd^ ± 1.48	7.98 ^def^ ± 1.37	$7.72{\;}^{\mathrm{gh}}\pm 1.62$
**S2**	$7.82{\;}^{j}\pm 1.51$	$7.50{\;}^{q}\pm 1.28$	$7.31{\;}^{t}\pm 1.32$	$7.12{\;}^{u}\pm 1.48$	$6.89{\;}^{v}\pm 1.56$	$7.54{\;}^{o}\pm 1.24$	$7.30{\;}^{p}\pm 0.89$	$7.18{\;}^{q}\pm 0.75$	$7.06{\;}^{r}\pm 1.16$	$6.78{\;}^{t}\pm 1.63$	$6.44{\;}^{m}\pm 1.26$	$6.32{\;}^{n}\pm 0.94$	$6.12{\;}^{q}\pm 0.81$	$5.90{\;}^{r}\pm 1.15$	$5.62{\;}^{s}\pm 1.54$	$7.0{\;}^{j}\pm 0.86$	$6.42{\;}^{l}\pm 1.1$	6.30 ^lmn^ ± 1.2	6.11 ^no^ ± 0.71	$6.04{\;}^{o}\pm 1.21$
**S3**	$8.09{\;}^{d}\pm 0.95$	$7.88{\;}^{i}\pm 0.84$	$7.63{\;}^{o}\pm 0.56$	$7.51{\;}^{q}\pm 0.77$	$7.34{\;}^{s}\pm 1.12$	$8.36{\;}^{a}\pm 1.16$	$8.26{\;}^{d}\pm 1.02$	$8.11{\;}^{g}\pm 1.22$	$7.95{\;}^{i}\pm 1.47$	$7.78{\;}^{l}\pm 1.1$	$6.98{\;}^{g}\pm 1.12$	$6.76{\;}^{k}\pm 1.1$	$6.42{\;}^{m}\pm 0.91$	$6.28{\;}^{o}\pm 1.30$	$6.14{\;}^{q}\pm 0.85$	$8.34{\;}^{\mathrm{ab}}\pm 0.86$	$8.22{\;}^{\mathrm{bc}}\pm 1.15$	8.08 ^cde^ ± 0.56	7.91 ^efg^ ± 1.28	$7.75{\;}^{\mathrm{gh}}\pm 0.98$
**S4**	$8.14{\;}^{b}\pm 0.86$	$8.06{\;}^{e}\pm 0.64$	$7.83{\;}^{j}\pm 0.91$	$7.71{\;}^{m}\pm 0.78$	$7.53{\;}^{p}\pm 1.16$	$8.28{\;}^{c}\pm 0.89$	$8.16{\;}^{f}\pm 0.96$	$8.07{\;}^{h}\pm 1.18$	$7.80{\;}^{k}\pm 1.25$	$7.68{\;}^{n}\pm 1.04$	$7.03{\;}^{f}\pm 1.08$	$6.81{\;}^{j}\pm 1.26$	$6.56{\;}^{l}\pm 1.45$	$6.30{\;}^{\mathrm{no}}\pm 1.23$	$6.19{\;}^{p}\pm 0.68$	$8.12{\;}^{\mathrm{cd}}\pm 0.94$	$7.96{\;}^{\mathrm{def}}\pm 0.72$	7.83 ^fgh^ ± 0.58	7.68 ^h^ ± 0.84	$7.44{\;}^{i}\pm 0.62$
**S5**	$8.20{\;}^{a}\pm 0.78$	$8.12{\;}^{c}\pm 0.86$	$8.04{\;}^{f}\pm 0.91$	$7.92{\;}^{g}\pm 0.64$	$7.80{\;}^{k}\pm 1.10$	$6.86{\;}^{s}\pm 1.34$	$6.62{\;}^{u}\pm 1.18$	$6.41{\;}^{v}\pm 1.27$	$6.16{\;}^{w}\pm 1.06$	$6.02{\;}^{x}\pm 0.89$	$7.26{\;}^{b}\pm 1.1$	$7.13{\;}^{d}\pm 1.28$	$7.04{\;}^{f}\pm 1.36$	$6.97{\;}^{g}\pm 1.12$	$6.88{\;}^{h}\pm 0.92$	$7.30{\;}^{i}\pm 1.16$	$6.94{\;}^{j}\pm 1.28$	6.70 ^k^ ± 1.34	6.51 ^mno^ ± 1.58	$6.32{\;}^{\mathrm{lm}}\pm 1.42$

Note: Different letters superscripted denote statistical difference (*p* < 0.05) within a column. The mean scores ± standard deviation are shown in the table. Nine-point hedonic scale: 1 = dislike extremely, 2 = dislike very much, 3 = dislike moderately, 4 = dislike slightly, 5 = neither like or dislike, 6 = like slightly, 7 = like moderately, 8 = like very much, and 9 = like extremely.
